# A core-level electron spectroscopy study of gas-phase 1,2,3-benzotriazole and methyl derivatives

**DOI:** 10.3762/bjnano.17.88

**Published:** 2026-09-18

**Authors:** Sven Bölke, Christophe Nicolas, John Bozek, Heiko Peisert, David R Batchelor

**Affiliations:** 1 Institut für Physikalische und Theoretische Chemie, Universität Tübingen, Auf der Morgenstelle 18, D-72076 Tübingen, Germany; 2 Synchrotron SOLEIL, L’Orme des Merisiers, Départementale 128, 91190 Saint-Aubin, France; 3 Karlsruhe Institute of Technology, Institute for Photon Science and Synchrotron Radaiation (IPS), Hermann-von-Helmholtz-Platz 1, D-76344 Eggenstein-Leopoldshafen, Germany

**Keywords:** Auger resonance, density functional theory, gas phase, near-edge X-ray absorption fine structure, tautomerism

## Abstract

A gas-phase photoelectron spectroscopy and X-ray absorption spectroscopy study of 1,2,3-benzotriazole and methyl derivatives was undertaken to investigate the influence of the tautomerism on the spectroscopic signature in greater detail. Soft X-ray photoelectron spectroscopy of the nitrogen core level, nitrogen K-edge near-edge X-ray absorption fine-structure measurements, and mapping of the resonant Auger spectroscopy were performed. High-energy ultraviolet photoemission spectroscopy was also undertaken to compare with the previous studies using low-energy low-energy Helium I and Helium II excitation. The gas-phase characterization is an essential step for interpreting adsorbed molecular phases and thin films on surfaces. The results also served as a test of STOBE density functional theory calculations, in particular the near edge.

## Introduction

Nanoparticles and their properties, such as large surface area, tailored surfaces, and catalytic activity, play an important role in pharmaceutical, medicinal chemistry [1] and also synthetic chemistry [2]. Understanding the adsorption either from the liquid or gas phase, and its bonding in adsorbed molecular phases or thin films is fundamental to nanoparticle research.

**Figure 1:**
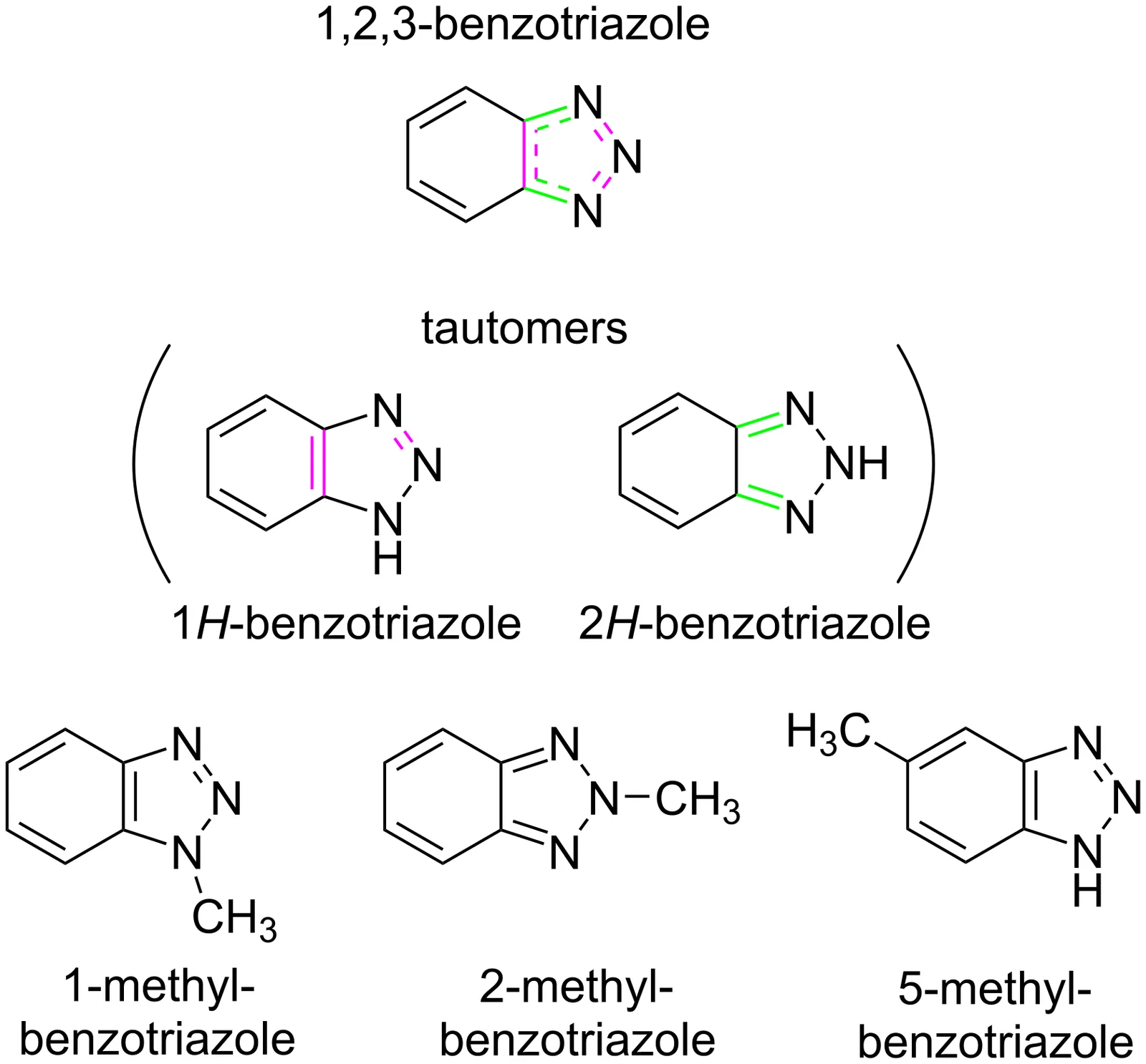
Molecular structures of 1,2,3-benzotriazole (BTAH), the two tautomers and the methyl derivatives 1-methylbenzotriazole (1-methylBTA), 2-methylbenzotriazole (2-methylBTA) and 5-methylbenzotriazole (5-methylBTA). For BTAH, delocalised bonding in the triazole ring is indicated by the coloured (and dashed) bonds, the hydrogen is omitted for clarity.

Benzotriazole (BTAH) is extensively used in diverse areas ranging from corrosion protection [3–5], synthetic chemistry [6–8], to pharmaceuticals, and medicine [9–11]. It displays a considerable variety in its adsorption behaviour on various surfaces, where hydrogen bonding can play an important role [12–13]. The material is a crystalline solid with a high degree of hydrogen bonding [14] and a high vapour pressure of 0.05 mbar at room temperature. In the gas phase, it has been demonstrated that the molecule exists in two tautomeric forms [15–16], 1H and 2H, shown in Figure 1.

The ratio of tautomers at 100 °C is not clear from either theoretical or experimental studies [15–16]. The energies for the optimised structures of the tautomers and dimers from STOBE calculations differ by a few hundred milli electron volts and suggest that a dimeric structure is relatively stable. Consequently, given the large hydrogen bonding in the solid and its high vapour pressure at room temperature, BTAH may also exist as hydrogen-bonded dimers [13] in the gas phase. This might also explain the complexity seen in the valence photoelectron spectra using helium excitation [16–18].

The gas-phase electronic structure and bonding in the molecule is necessary to understand the adsorbed molecular phases and thin films on surfaces [19–20]. The electronic structure and bonding of BTAH is the theme of this study. Four molecules 1,2,3-benzotriazole, 5-methylbenzotriazole, 1-methylbenzotriazole, and 2-methylbenzotriazole, shown in Figure 1, were studied. The methyl derivatives were used to mimic the experimental nitrogen core-level spectra of the 1H and 2H tautomers without hydrogen bonding. Although studied, results for 5-methylbenzotriazole are not shown as they did not significantly differ from that of 1,2,3-benzotriazole. Core-level photoelectron spectroscopy (PES), ultraviolet phototoelectron spectrocopy (UPS), and near-edge X-ray absorption fine-structure (NEXAFS) spectroscopy measurements were performed at the PLEIADES beamline of the synchrotron SOLEIL (Paris, France). To substantiate the interpretation of the spectra, density functional theory (DFT) calculations were performed using the STOBE code [21]. The DFT calculations also served as a basis for using the core-level spectra of the methyl derivatives for modelling those of the hydrogen tautomers.

The energy range of the PLEIADES beamline enabled UPS measurements at higher photon energies, which are compared to previous studies [16–18] using He I,II (*h*ν 21.23 eV, *h*ν 40.80 eV) excitation. Near-edge X-ray absorption fine-structure spectroscopy is sensitive to changes in the unoccupied valence orbitals, and this was exploited in a previous study to illustrate hydrogen bonded polymerisation of benzotriazole on Au(111) and Cu/Au(111) surfaces [12–13]. The problems with possible assignment of the previous UPS was significant in choosing the atomic specificity of core-level spectroscopy with its relatively simpler interpretation.

## Results and Discussion

### Core-level PES, NEXAFS, and UPS

The study concentrated on the methyl derivatives of benzotriazole (see Figure 1) as tautomerism is absent in these molecules (except for 5-methylbenzotriazole). Core-level electron spectroscopy was chosen due to its specificity in identifying spectral features compared to the earlier UPS studies [16–18]. The UPS valence spectra of the earlier work of 1-methyl and 2-methylbenzotriazole, although similar to that of 1,2,3-benzotriazole, displayed differences that might be attributed to other bonding, and/or, to the calculation of the valence structure and multi-electron excitations. Such difficulties do not exist for the core-level spectroscopy, being relatively straightforward in assignment.

**Figure 2:**
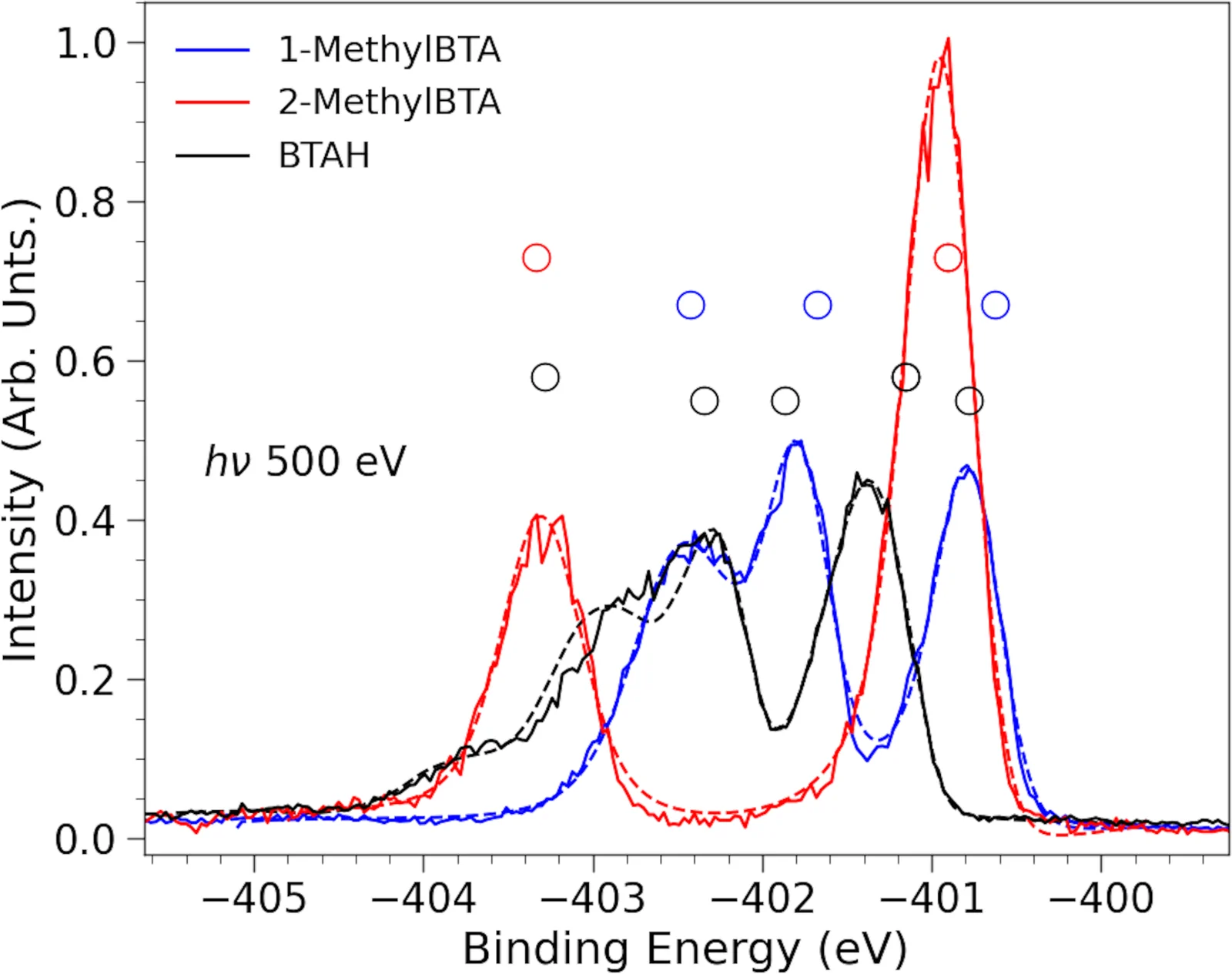
Nitrogen core-level photoelecltron spectra for 1-methylbenzotriazole, 2-methylbenzotriazole, and 1,2,3-benzotriazole. The open circles are the binding energies from STOBE calculations. 5-Methylbenzotriazole is omitted as there is little diﬀerence with BTAH.

Figure 2 shows the area-normalised nitrogen core-level photoemission using soft X-ray energy photons of 500 eV for 1-methylbenzotriazole, 2-methylbenzotriazole, and 1,2,3- benzotriazole. The spectra are as expected. Two peaks (two different chemical environments, Figure 1) are visible for the symmetric 2-methylbenzotriazole and three peaks are visible for the asymmetric 1-methylbenzotriazole (three different chemical environments, Figure 1). The 1,2,3-benzotriazole spectrum can be seen to be resolved into a shifted (0.45 eV) two-component admixture of the above spectra: Four peaks are seen as two peaks with binding energy of ≈401 eV overlap. To confirm this and to investigate further STOBE [21] calculations of the two methyl derivatives and the two tautomers were performed. The red and blue open circles in Figure 2 represent the calculated nitrogen core-level binding energies of the two methyl derivatives, and the black circles for the 1*H* and 2*H* tautomers (vertically separated). The binding energies were shifted due to the vacuum level (work function of the analyser), and a relaxation shift was included which is not accounted for in the DFT calculations. The dashed lines are fits of an “asymmetric” Voigt profile (see Supporting Information File 1).

Asymmetry in the peaks is clearly visible and reflects the vibrations, rotational contributions, post-collision interactions [22–23], and other inelastic losses (here it is the vibrations [24]). The stoichiometry obtained from the fit of the spectra with the “asymmetric” Voigt yielded the expected atom ratios for the methyl derivatives. The black dashed line for BTAH is the admixture of fitting the methyl spectra (red and blue curves), and it has a small shift. The shift (0.45 eV) is ascribed to a differing relaxation present in the interchanging mixture of the two tautomers compared to that of the static molecules. The results of the fit give a mixture of 15.6% for the 2*H*-benzotriazole and 84.4% for the 1*H*-benzotriazole (1H-BTA/2H-BTA ratio 5.41 ± 9%), and does not agree with the vibrational spectroscopy study of Roth [15]: A question as to the relative stability of the two tautomers existed on account of calculations [15–16].

**Figure 3:**
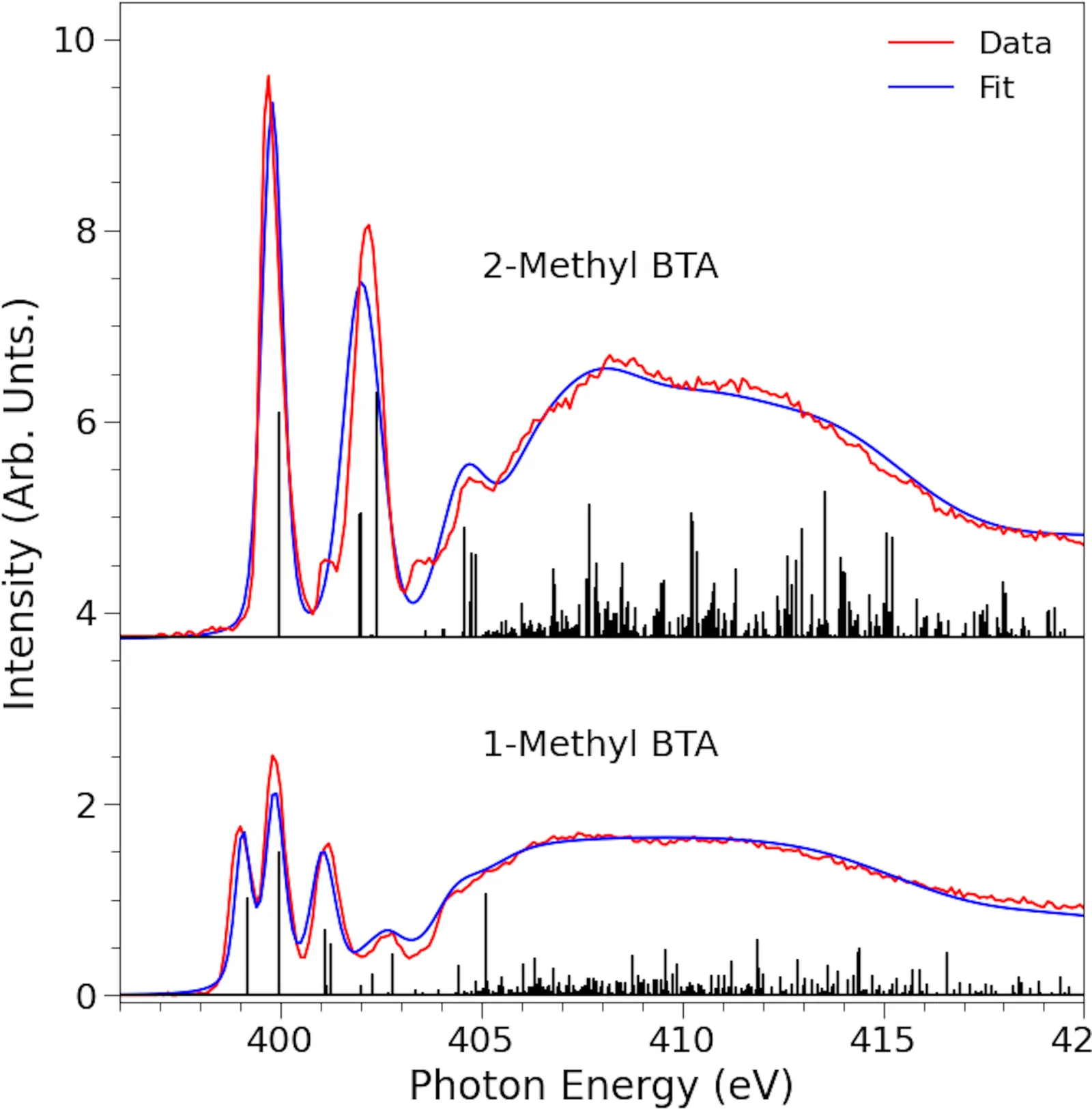
Nitrogen K-edge NEXAFS for 1-methylbenzotriazole and 2-methylbenzotriazole. The blue curves are fits to the experimental spectra as described in the text. The transitions are depicted as bars in the plot.

The NEXAFS of the 1-methyl and 2-methyl derivatives are shown in Figure 3 (spectra are normalised to the post-edge *h*ν 420 eV) together with the fits of calculations to the spectra using STOBE [21] and the calculated oscillator strengths (bars in figure) with a varying peak width [25]. The variable width takes into account the decreasing monochromator resolution with photon energy, detector window, and larger lifetime broadening due to the increased decay channel density as the energy is increased.

As with the photoelectron spectra, an “asymmetric” Voigt function (see Supporting Information File 1) was used. The fits are good showing the physics of the model, and the DFT calculation describes the gas-phase experimental spectra well.

**Figure 4:**
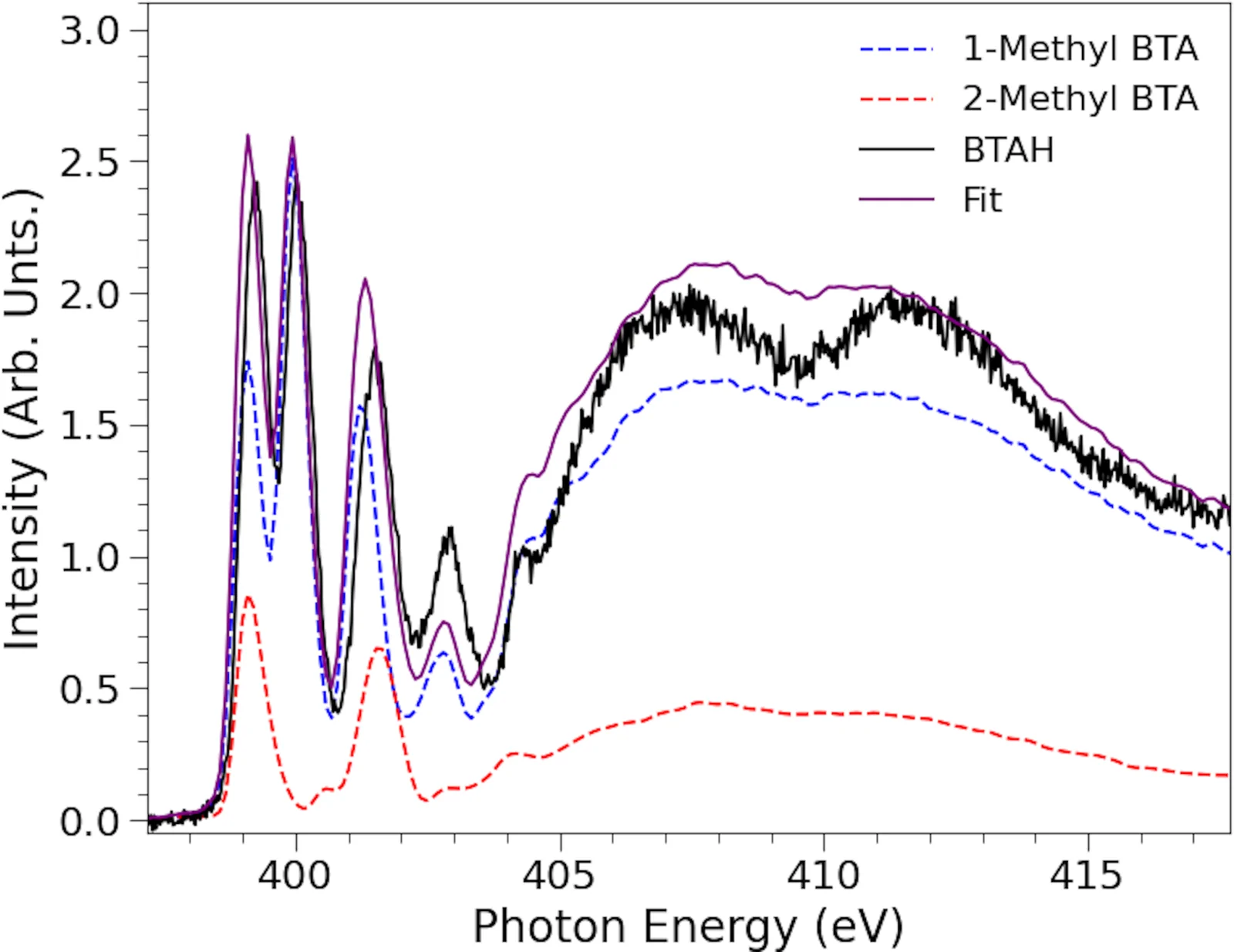
Modelling of the BTAH NEXAFS spectrum using the experimental 1-methylbenzotriazole and 2-methylbenzotriazole spectra: STOBE calulations of the 1*H* and 2*H* tautomers showed negligible difference with the methyl derivative spectra. The curves for the methyl derivatives are smoothed to remove noise but preserve sharp features of the resonance, and represent actual contributions.

The spectrum for 1,2,3-benzotriazole (Figure 4) shows that the principal features of both methyl spectra are also present. To compare the core-level photoelectron spectroscopic results for the tautomeric ratio with that of the 1,2,3-benzotriazole NEXAFS, the BTAH spectrum needs to be modelled. Although, the STOBE calculations and above fitting are good, to fit the experimental BTAH spectrum with a mixture of the methyl experimental spectra is a better approach: STOBE nitrogen-edge NEXAFS calculations for the 1*H*-benzotriazole and the 2*H*-benzotriazole also showed little difference with that of the methyl derivatives. The result of the fit (purple curve) is in good agreement with the experimental data. The fit yields 15% (red dashed curve) for the 2*H* tautomer and 85% (blue dashed curve) for the 1*H* tautomer (1H-BTA/2H-BTA ratio 5.66 ± 21%). This is in good agreement with the core-level photoemission fitting results above, and does not agree with the vibrational study of Roth [15].

Photoelectron spectroscopy is a direct measure of the populations of the 1*H* and 2*H* tautomers compared to that deduced from the vibrational intensities with temperature by Roth [15]. The van’t Hoff isochore plot of Roth [15] is linear over the range studied (386–503 K) indicating its validity, but yields a Δ*Η*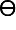 too low to give the above population for a reasonable temperature of the cell. This might reflect that the equilibrium is changing in the lower region of the above range, and/or that unlike the Roth work the cell is not in thermal equilibrium and the temperature of the gas probed by the X-rays is uncertain. This may be the case as the cell is pumped by the main chamber. The probe by X-rays or infrared is considerably much faster than the inter conversion rate of the two tautomers. The above result for the tautomeric ratio and the STOBE modelling indicate that no other bonding [12] is seen in this low-pressure gas-phase study of BTAH.

**Figure 5:**
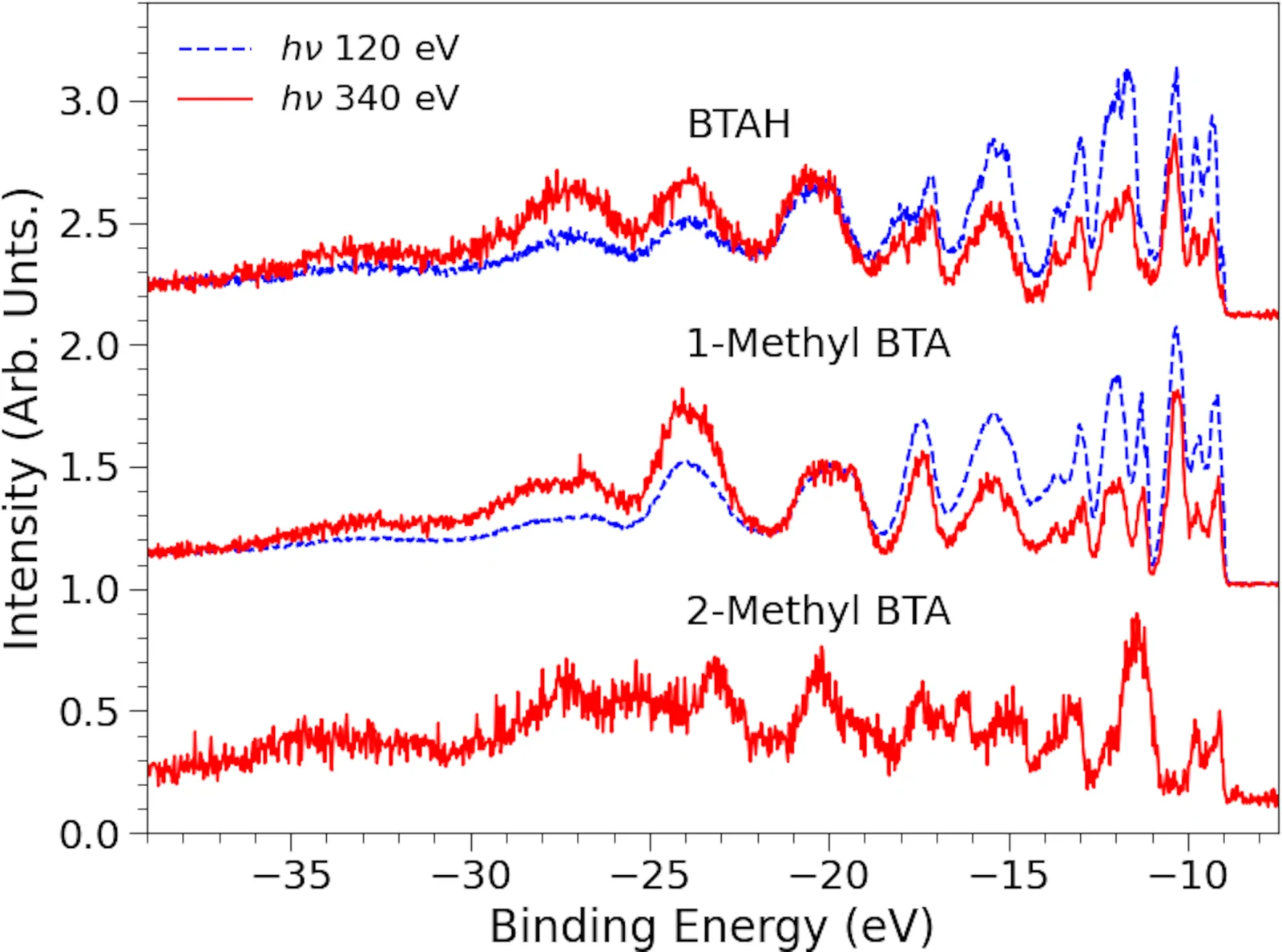
Valence ultraviolet photoemission spectra for BTAH, 1-methylbenzotriazole and 2-methylbenzotriazole measured with photon energies of 120 and 340 eV. The 120 eV energy spectrum for 2-methylbenzotriazole is not shown due to a small water contamination.

As stated above, the UPS studies of Rademacher, Novak, and Leupin [16–18] were a motivation for the present experiment at PLEIADES as resolution and flux at both low and high energy far exceeded that of the previous studies. The UPS using 120 and 340 eV soft X-rays are shown in Figure 5 for the methyl derivatives and 1,2,3-benzotriazole (the 120 eV spectrum for 2-methylBTA is not shown due to a small water contamination). The higher-energy 340 eV spectra show a significant (expected) broadening in comparison to that of the lower-energy spectra. They are in reasonable agreement with previous studies [16–18] but differ principally in signal to noise and energy range. A simple admixture of the methyl spectra to obtain the BTAH spectrum and the tautomeric ratio was not successful presumably due to differing methyl contributions and other excitations in this region. STOBE calculations were not successful in fitting of the spectra. The calculation is beyond the scope of this study and requires calculating multi-electron excitations as described below.

### Resonant Auger spectroscopy and photon-energy/electron-energy maps

Resonant Auger spectroscopy (RAS) has been used to probe inter- and intramolecular charge transfer dynamics in femto- and atto-second time scales in thin films and at interfaces [26–29]. The spectra contain information about the delocalization of electronic states and the local nature of the excitation at a particular atom and allow the assignment of contributions in the valence region. This was also undertaken as differences in bonding, and excitations should result in additional structures being visible in the valence region which is not seen in the NEXAFS [12]. The data space is large as it involves both the electron kinetic energy and the photon energy. Typically, in such studies, a resonance in the NEXAFS is chosen and a photoelectron spectrum is taken covering both the valence region and the Auger peak. The beamline PLEIADES has sufficient photon flux and an analyser with a large collection angle, so that spectral maps [30] of photon energy versus electron binding energy (or kinetic energy) can be generated, making it easier to distinguish details, such as the spectator and participator trends, and help to identify and distinguish multi-electron excitations in the valence region.

**Figure 6:**
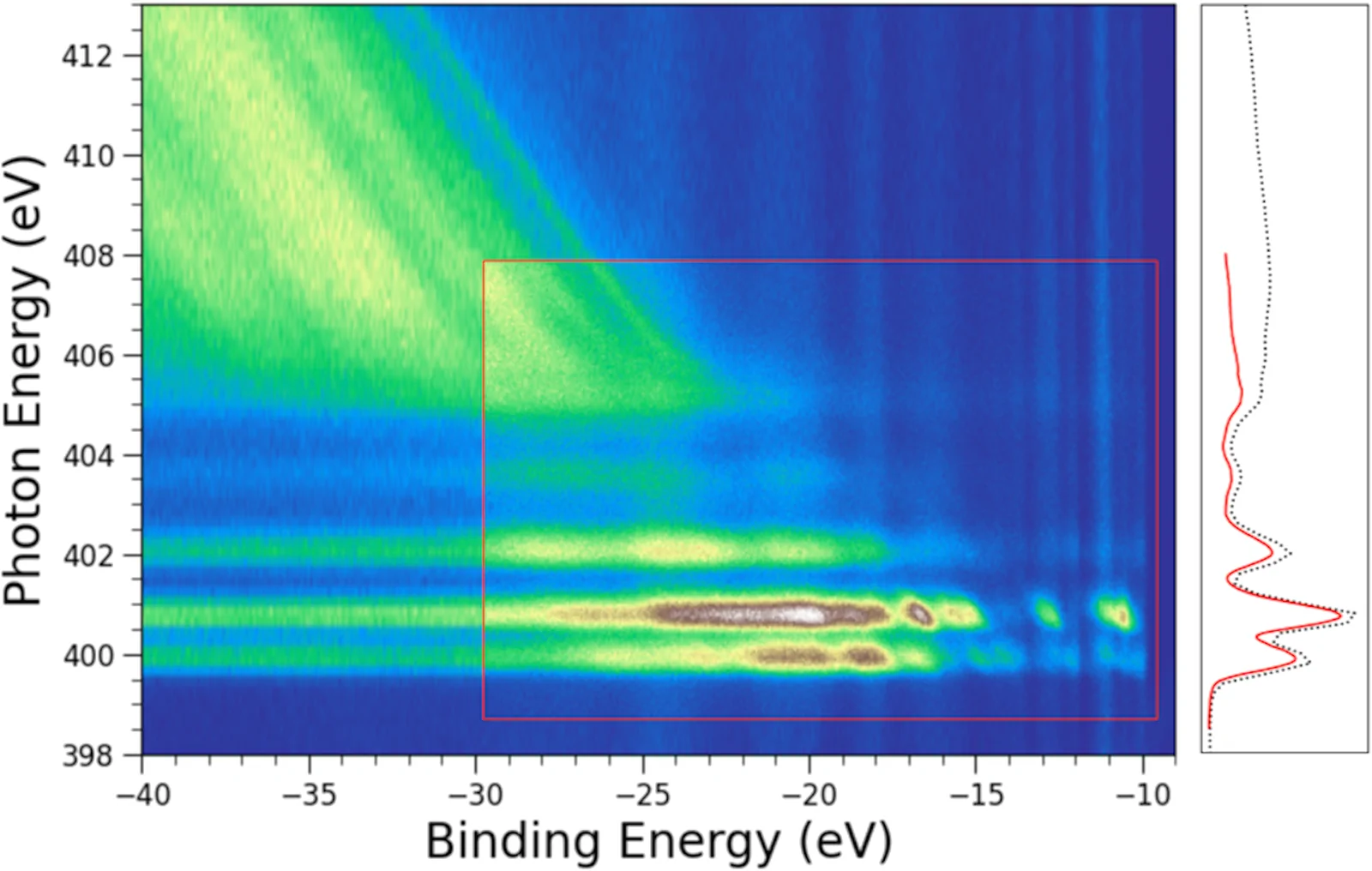
Shows a large-range Auger resonance map of the NEXAFS and Auger region. Sharp peaks (shoulders), also constant in kinetic energy, are visible on the broad Auger peak. The inset shows a detailed map scan of the NEXAFS region clearly showing peaks on resonance in the valence region, demonstrating both Auger- and photoelectron-like behaviour. The spectra shown on the right are the NEXAFS after integrating the two regions.

Such a map is shown for 1-methylbenzotriazole in Figure 6 and displays the photoemission and Auger transition as a function of photon energy and electron binding energy. Below the nitrogen K edge in the pre-edge region, the spectra simply reflect the valence structure albeit with considerable broadening compared to the valence spectra measured with lower photon energies.

As the photon energy is increased, a resonant structure appears in the valence region with increased intensity for certain transitions. A detailed region is shown in the inset. Looking closely, some of the resonances appear to disperse, decrease with photon energy (constant kinetic energy) are Auger like and/or, behave as photoelectrons (constant binding energy). The resonances are attributed to the participator and spectator excitations: there is interference involved as before and after resonance the intensity is comparable but on resonance it is an order of magnitude larger.

In addition, a broad peak can be identified which is due to spectator autoionisation (see below). Above the edge, the Auger peak is clearly visible but is shifting in kinetic energy due to the post-collision interaction [22–23] before remaining constant in energy. On the right handside of the maps in Figure 6 are the NEXAFS spectra obtained by integrating the signal with binding energy for the wide scan and the detailed region of the inset. The spectra differ due to the different contributions of the Auger transition (kinetic energy of 374.2 eV).

**Figure 7:**
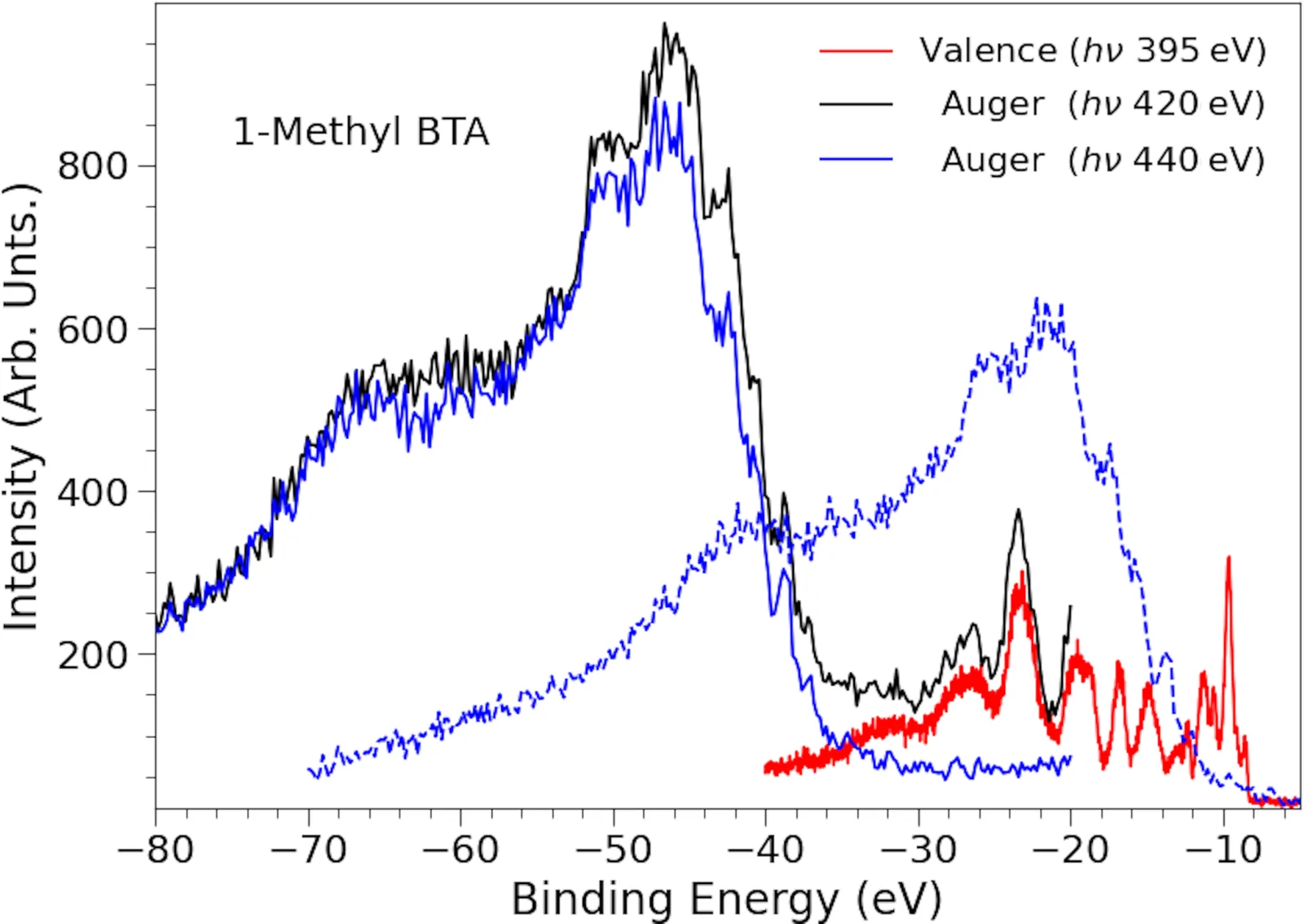
Valence region just below the NEXAFS region and Auger spectra taken above the edge showing sharp shoulders on the Auger peak. See text for a more detailed description.

On closer inspection, relatively sharp shoulders (kinetic energies of 377.55, 379.45, and 381.35 eV) can be discerned on the Auger peak, which are also constant in kinetic energy. These are attributed to Auger lines involving the very outer valence electrons and are long lived similar to those seen for molecular nitrogen [31–34]. This is shown in detail in Figure 7, which shows the Auger excitation for photon energies of 440 and 420 eV together with a valence spectrum (*h*ν 395 eV). The Auger spectrum taken at 440 eV has been shifted and scaled to lie on top of the 420 eV spectrum. The blue dashed line is the spectrum shifted and scaled to a hypothetical position for photons of 395 eV, and lies on top of the valence region. It is clearly seen that the sharp shoulders are in the valence region, indicating that they are a two-valence (multi-electron) electron excitation.

The valence occupied orbital structure is insufficient to explain the valence spectra and requires other contributions, excitations. The simplest is to include excitations to the unoccupied states of the molecule. A simple model was used to calculate the Auger excitation and additional valence excitation, satellites – see Supporting Information File 1 and Figure S1. The model shows a broad spectrum of possible one electron excitations into the valence region and above, and the two electron excitations reproduce the position and envelope of the Auger transition. However, the simple model is unable to calculate the detailed structure as this requires calculation of the transition probabilities and interference of the various states, which is outside the scope of this experimental study. The availability and energy of the satellite excitations indicates that these features are also present at low photon energy.

**Figure 8:**
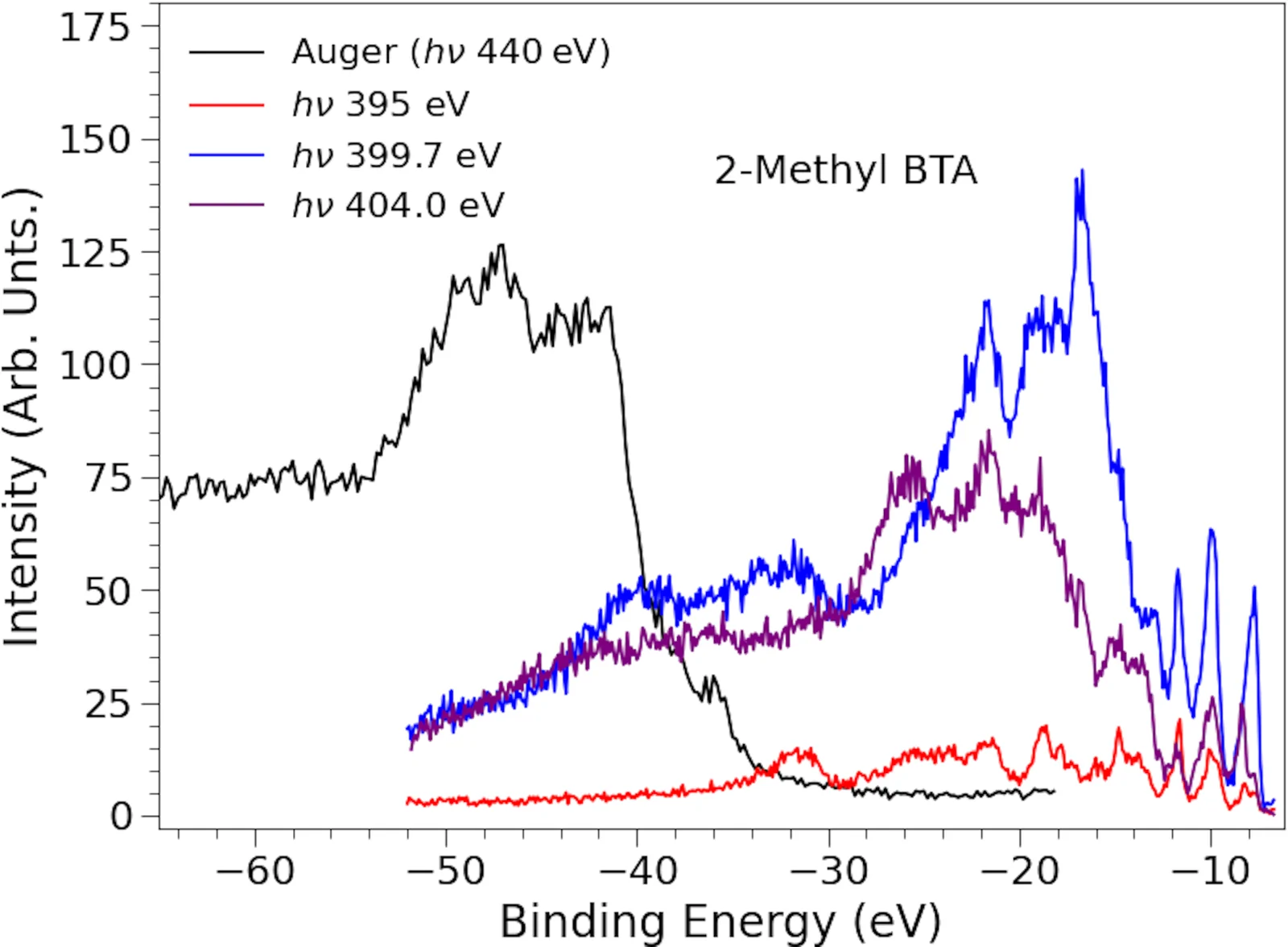
Resonance spectra for 2-methylbenzotriazole, valence region, and Auger region. See text for a more detailed description.

Figure 8 shows scans for the two resonances (399.7 and 404.0 eV) of 2-methylbenzotriazole, valence region (*h*ν 395 eV) and the Auger region (*h*ν 440 eV). The two resonant spectra show that a large number of excitations are present in the valence region as is indicated by the simple model (Support Information File 1). Also seen are broad peaks which resemble the Auger peak and result from autoionisation of the spectator excitations. The resulting Auger transitions are shifted in energy due to screening by the spectator electron. The calculation of these spectra is beyond the scope of this work [24].

## Conclusion

A core-level spectroscopic study of BTAH and its methyl derivatives was undertaken to characterise the gas phase, investigate the exhibited tautomerism, and the electronic structure of the molecules. This is a necessary step to understand the adsorbed molecular phases and thin films on surfaces. The STOBE DFT calculations explained well the experimental core-level photoemission and NEXAFS of the gas phase. The maps and Auger resonance spectra show a rich, diverse, and complex behaviour illustrating the spectator, participator (satellite structures), and post-collision interactions. The calculations, however, could not explain the UPS or Auger resonance structure. More detailed calculations are required including interference of the various states and multi-electron transitions, the subject of a separate work. However, it is evident that one-electron and two-electron excitations are responsible for additional structure in the valence region and are also present at low-photon energy excitation.

## Experimental

The experiments were carried out at the PLEIADES [24,35] beamline at the French synchrotron SOLEIL (Paris). The monochromator is a variable line-spaced (VLS) and variable groove-depth (VGD) plane-grating monochromator (PGM) without an entrance slit using an Apple II permanent magnet undulator. The measurement chamber is equipped with a VG-SCIENTA R4000 WAL electrostatic spherical energy analyser. NEXAFS was measured using the nitrogen Auger electron transition. This was used instead of the partial electron yield, or total electron yield detection as the signal from the chamber residual gas background along the synchrotron light path dominated the signal (strong π–π* resonance – Prince, Feiffel [36–37]). As a consequence, the signal from the light interaction region of the in situ cell and the analyser focus was used avoiding nonfocussed contributions.

The vapour pressure of the liquid 2-methylbenzotriazole is sufficiently high at room temperature to allow direct dosing for the experiment. Further purification of 2-methylbenzotriazole was performed by freeze/thaw cycles. For the other molecules, an in situ evaporation cell was used to achieve a high enough pressure. The conditions and temperatures for the steady-state operation of the cell was found by careful gradual heating while monitoring the pressure, temperature, and signal. This avoided sudden gas bursts during the analyser operation. Differential pumping was needed to obtain a high enough gas pressure at the cell focus and was achieved through increased length of the tubes facilitating the entrance and exit of the X-rays to the cell. This ensured that the background analyser pressure stayed below 1 × 10^−6^ mbar. Nitrogen NEXAFS was obtained using the nitrogen Auger peak measured with a high analyser pass energy and a kinetic energy to maximise the signal-to-noise ratio and avoid including the direct photoelectron signals.

All of the methyl-substituted benzyotriazoles and BTAH of sufficient purity could be bought commercially apart from the 2-methylbenzotriazole. This molecule was successfully synthesised by AK Bräse at the Institut für Organische Chemie at the Karlsruher Institut für Technologie (KIT) for the beamtimes.

The binding energies for the core photoemission and NEXAFS calculations used for the fitting procedure of the data were calculated by using the DFT program STOBE [21] as described in the results and discussion section and in Supporting Information File 1. The structures were energy optimised and the bond lengths agreed with that found in other studies [16].

## Supporting Information

File 1 Additional information.

## Data Availability

Data generated and analyzed during this study is available from the corresponding author upon reasonable request.
