# Supplementary material for: A core-level electron spectroscopy study of gas-phase 1,2,3-benzotriazole and methyl derivatives
Source: Beilstein J Nanotechnol. 2026 Sep 18;17:1282–9. doi: 10.3762/bjnano.17.88 (PMC13591422; doi:10.3762/bjnano.17.88)
Supplement: Supplementary file 1 [file Beilstein_J_Nanotechnol-17-1282-s001.pdf]

BEILSTEIN JOURNAL OF NANOTECHNOLOGY

Supporting Information

for

A core-level electron spectroscopy study of gas-phase 1,2,3-benzotriazole and methyl derivatives

Sven Bölke, Christophe Nicolas, John Bozek, Heiko Peisert and David R. Batchelor

Beilstein J. Nanotechnol. **2026,** *17,* 1282–1289. doi:10.3762/bjnano.17.88

Additional information

License and Terms: This is a supporting information file under the terms of the Creative Commons Attribution License (https://creativecommons.org/licenses/by/4.0). Please note that the reuse, redistribution and reproduction in particular requires that the author(s) and source are credited and that individual graphics may be subject to special legal provisions.
The license is subject to the *Beilstein Journal of Nanotechnology* terms and conditions: (https://www.beilstein-journals.org/bjnano/terms)

"Asymmetric" Voigt line shape

The line shape is based on a Voigt with the sigma of the Gaussian peak contribution as an error function between two limits.

$$\boldsymbol{\sigma(x)=\sigma_{av}+\sigma_{df}/2-\sigma_{df}\left[1-Erfc\left(\frac{x}{\sigma_g}\right)/2\right]}$$

the shift in the peak maximum is given by

$$\boldsymbol{\Delta x_{shf} \approx \frac{\sqrt{\pi}\sigma_g\sigma_{df}\sigma_{av}}{\pi{\sigma_g}^2+2{\sigma_{df}}^2}}$$

where $\boldsymbol{\sigma_{df}}$ is the difference and $\boldsymbol{\sigma_{av}}$ the average of the Gaussian limits, $\boldsymbol{\sigma_a}$, $\boldsymbol{\sigma_b}$ and $\boldsymbol{\sigma_g}$ is the broadening of the error function.

As it is based on a Voigt peak, the area is unity.

The value of physical parameters related to the vibrations, rotations, post-collision interactions, other losses to the asymmetry is model dependent and may be difficult to extract due to combined losses. Here, the line shape was chiefly used to determine the area and stoichiometry. A Gaussian convolution is intrinsic to the Voigt line shape. The width can be estimated from the Voigt full width half maximum.

Benzotriazole and methyl derivatives

1,2,3-Benzotriazole, white crystalline solid RT, melting point 98 Celsius

1-methylbenzotriazole, white crystalline solid RT, melting point 64 Celsius

2-methylbenzotriazole, pale yellow liquid RT

5-methylbenzotriazole, white crystalline solid RT, melting point 82 Celsius

All chemicals were from TCI or Aldrich (98%), apart from the 2-methylbenzotriazole which was synthesised by AK Bräse, at the Institut für Organische Chemie

Valence region, excitations (satellites), and Auger

The Figure S1 shows the occupied valence levels (red curve) for 1-methylbenzotriazole and a simple model for one-electron excitations to the unoccupied states and two-electron hole states for the Auger transition using the calculated STOBE occupied and unoccupied levels. The spectra were simply Gaussian convoluted to make the transition number and position clearer. The one-electron excitations (black curve) show a broad distribution in the valence region from ≈10 eV and extending well above. The two-electron excitations roughly reproduce the width and envelope of the observed Auger excitation.

A proper calculation of the Auger transition and valence excitations is beyond the scope of this study.

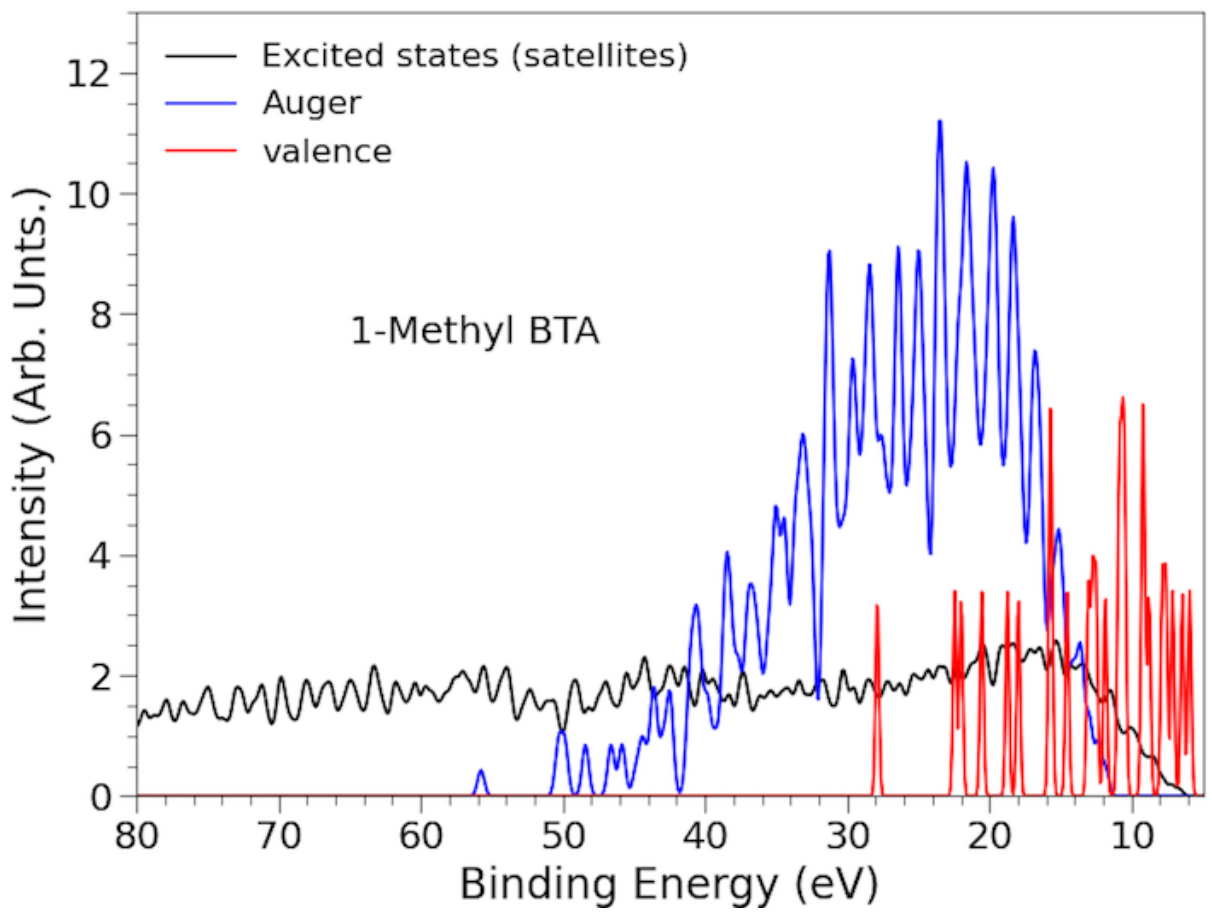

Figure S1: Occupied valence states and simple calculation of Auger and valence electron excitations.

NEXAFS fitting

As previously described, the broadening and fit use a Voigt function with variable width [1,2]. The width models the decrease of the lifetime of the excitations as the decay channel probability increases above the edge, combined with other Gaussian broadening terms such as vibrations, monochromator resolution, and the detector window (see NEXAFS in Figure 6 of the main manuscript). Below the edge, the width linearly varies and above a power law is used. The core-edge and an energy scaling term are also free parameters in the fit. In this case the “asymmetric” Voigt is used.

References

1. Batchelor, D. R.; Aygül, U.; Dettinger, U.; Ivanovic, M.; Tournebize, A.; Mangold, S.; Forster, M.; Scherf, U.; Peisert, H.; Chassé, T. *Eur. Polym. J.* **2016,** *8,* 686–693. doi:10.1016/j.eurpolymj.2016.04.005
2. Grillo, F.; Batchelor, D.; Larrea, C. R.; Francis, S. M.; Lacovig, P.; Richardson, N. V. *Nanoscale* **2019,** *11,* 13017–13031. doi:10.1039/C9NR04152D
